# Diversification rates and phenotypic evolution in venomous snakes (Elapidae)

**DOI:** 10.1098/rsos.150277

**Published:** 2016-01-20

**Authors:** Michael S. Y. Lee, Kate L. Sanders, Benedict King, Alessandro Palci

**Affiliations:** 1 Earth Sciences Section, South Australian Museum, North Terrace, Adelaide, SA 5000, Australia; 2 School of Biological Sciences, University of Adelaide, Adelaide, SA 5005, Australia; 3 School of Biological Sciences, Flinders University, PO Box 2100, Adelaide, SA 5001, Australia

**Keywords:** macroevolution, body size, speciation rates, phylogenetics, reptiles

## Abstract

The relationship between rates of diversification and of body size change (a common proxy for phenotypic evolution) was investigated across Elapidae, the largest radiation of highly venomous snakes. Time-calibrated phylogenetic trees for 175 species of elapids (more than 50% of known taxa) were constructed using seven mitochondrial and nuclear genes. Analyses using these trees revealed no evidence for a link between speciation rates and changes in body size. Two clades (*Hydrophis*, *Micrurus*) show anomalously high rates of diversification within Elapidae, yet exhibit rates of body size evolution almost identical to the general elapid ‘background’ rate. Although correlations between speciation rates and rates of body size change exist in certain groups (e.g. ray-finned fishes, passerine birds), the two processes appear to be uncoupled in elapid snakes. There is also no detectable shift in diversification dynamics associated with the colonization of Australasia, which is surprising given that elapids appear to be the first clade of venomous snakes to reach the continent.

## 1. Introduction

Recent studies investigating the relationship between speciation rates and morphological evolution have produced contrasting results. In the extant biota, a link between elevated rates of speciation and morphological evolution would be reflected in highly speciose lineages exhibiting greater morphological disparity; conversely, groups with low species diversity should exhibit less disparity. Phylogenetic tests of this association have suggested a positive correlation between rates of speciation and phenotypic evolution in passerine birds [1] and ray-finned fishes [2], but no such association was found in mammals [3] and New World squamates [4], while both patterns have been proposed in plethodontid salamanders [5,6].

Large phylogenies with dense taxon sampling can improve estimates of diversification rates; however, extant diversity provides only limited information about speciation rates across deep time. Diversity patterns might be partly driven by differential extinction rather than speciation, and extinction rates are difficult to reconstruct from phylogenies of living taxa [7]. Low current diversity could be the result of extinction causing the decline of a previously speciose group, as in coelacanths and horses, which were much more diverse in the Triassic and Miocene, respectively [8,9]. The potential confounding factor of extinction is likely to be less problematic in molecular phylogenies of young, rapidly radiating groups such as that presented here, due to the fast net diversification of these clades, and the potentially high fraction of total species diversity that still survives.

We tested the proposed correlation between speciation rates and rates of phenotypic evolution in Elapidae, which represents the largest clade of highly venomous snakes and contains many of the most medically important species (e.g. [10,11]). Elapid snakes are a relatively young group (less than 40 Ma old), exhibiting some of the highest net diversification rates in reptiles [12]. This speciose clade (more than 350 known species; www.reptile-database.org) also shows a wide range of variation in adult body shape [13] and body size (e.g. 20 cm total length in *Simoselaps minimus* [14] and 585 cm in *Ophiophagus hannah* [15]). This makes it an excellent group to evaluate the possible relationship between speciation rates and rates of body size evolution. Following recent studies (e.g. [2,4]), we consider body size a suitable proxy for phenotypic variation because (i) body size is generally tightly correlated with shape and life-history traits in snakes (e.g. [16]) and organisms in general (e.g. [4,5,17]), (ii) it is one of the easiest morphological traits to quantify, and (iii) the relevant data are readily available in the literature for nearly all species of interest.

## 2. Material and methods

### 2.1 Molecular data and alignment

Molecular data for 175 species of elapids were obtained using the best-sampled genes for this group: mitochondrial 12S, 16S, ND4 and cytochrome *b*, and nuclear C-mos, RAG-1 (two regions) and RAG-2. The concatenated elapid sequences in Pyron *et al.* [18] were supplemented with those of additional marine taxa obtained from a study of seasnakes [19], and GenBank sequences for species of *Micrurus*, *Vermicella*, *Simoselaps*, *Brachyurophis* and *Oxyuranus*. New sequences were obtained for *Kolpophis annandalei* (16S, ND4, cytb) and for *Aipysurus foliosquama* (16S, cytb), *Hydrophis bituberculatus* (ND4) and *Hydrophis torquatus* (ND4), using the protocols in Sanders *et al.* [19]. GenBank accession numbers for all added sequences are in the electronic supplementary material, table S1.

Alignment was performed using MUSCLE [20] in Geneious Pro v. 5.1.7 [21]. The alignment of Pyron *et al*. [18] was used as the (preserved) core profile, to which additional sequences (see above) were added. Examination of the final resultant alignment revealed that the 12S and 16S alignments had anomalous regions, which were refined by eye or excluded from analysis. The final alignment consisted of 175 terminal taxa and 9083 base pairs (bp), of which 58 bp of RNA (12S and 16S) were excluded due to ambiguous alignment. The full alignment, including partition and exclusion sets, is in Dryad File 1 (doi:10.5061/dryad.cr788).

### 2.2 Phylogenetic analyses

The appropriate partitioning scheme and substitution models were selected using PartitionFinder [22]. From a total of 20 candidate partitions (12S, 16S, and codons 1, 2 and 3 for each of the six coding regions), the Bayesian information criterion selected a seven-partition scheme with the following substitution models: (i) nuclear coding regions, codons 1+2—HKYig; (ii) nuclear coding regions, codon 3—HKYg; (iii) mitochondrial coding regions, codon 1—GTRig; (iv) mitochondrial coding regions, codon 2—GTRig; (v) mitochondrial coding regions, codon 3—GTRg; (vi) 12S rRNA—GTRig; and (vii) 16S rRNA—GTRig.

Phylogeny and divergence dates were inferred simultaneously with MrBayes v. 3.2 [23], using the above partitions and models. All sequences were concatenated for analysis; multispecies coalescent methods were not implemented due to the small number of independent loci (mtDNA and three nuclear genes) and low variability of the nuclear genes used. Four nodes in the tree were calibrated, with hard minima and soft 95% maxima based on the fossil record (electronic supplementary material, table S2); these calibrations are broadly consistent with molecular estimates of these nodes (e.g. [24]). Stepping-stone analyses [25] favoured the uncorrelated igr relaxed clock (marginal likelihood −97111.59), over both the autocorrelated tk02 relaxed clock (−97279.75) and the strict clock (−97236.45).

**Figure 1.**
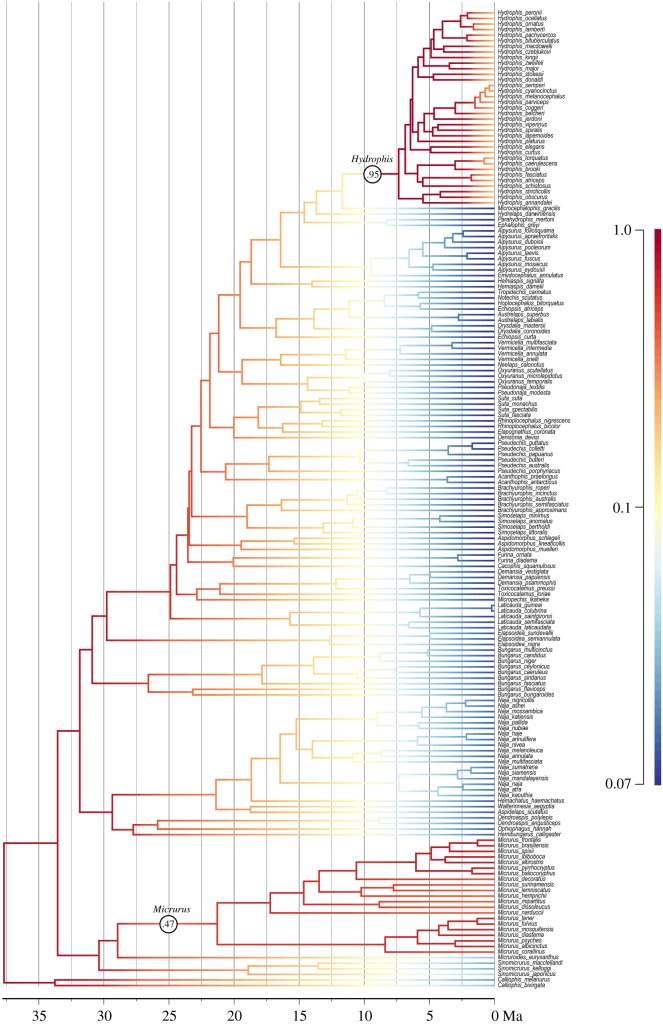
Datedphylogenetic tree of elapids, with rates of speciation inferred using BAMM (warmer colours = faster rates; scale bar on right represents speciation rate per Ma). Circled numbers indicate the two branches with the highest probability of rate shifts, i.e. *Hydrophis* and *Micrurus*. Tree with posterior probabilities of each clade is shown in electronic supplementary material, figure S1, rates of extinction are shown in electronic supplementary material, figure S4, and the nine rate shift configurations (for diversification) in the 95% credibility shift set are shown in electronic supplementary material, figure S5.

**Figure 2.**
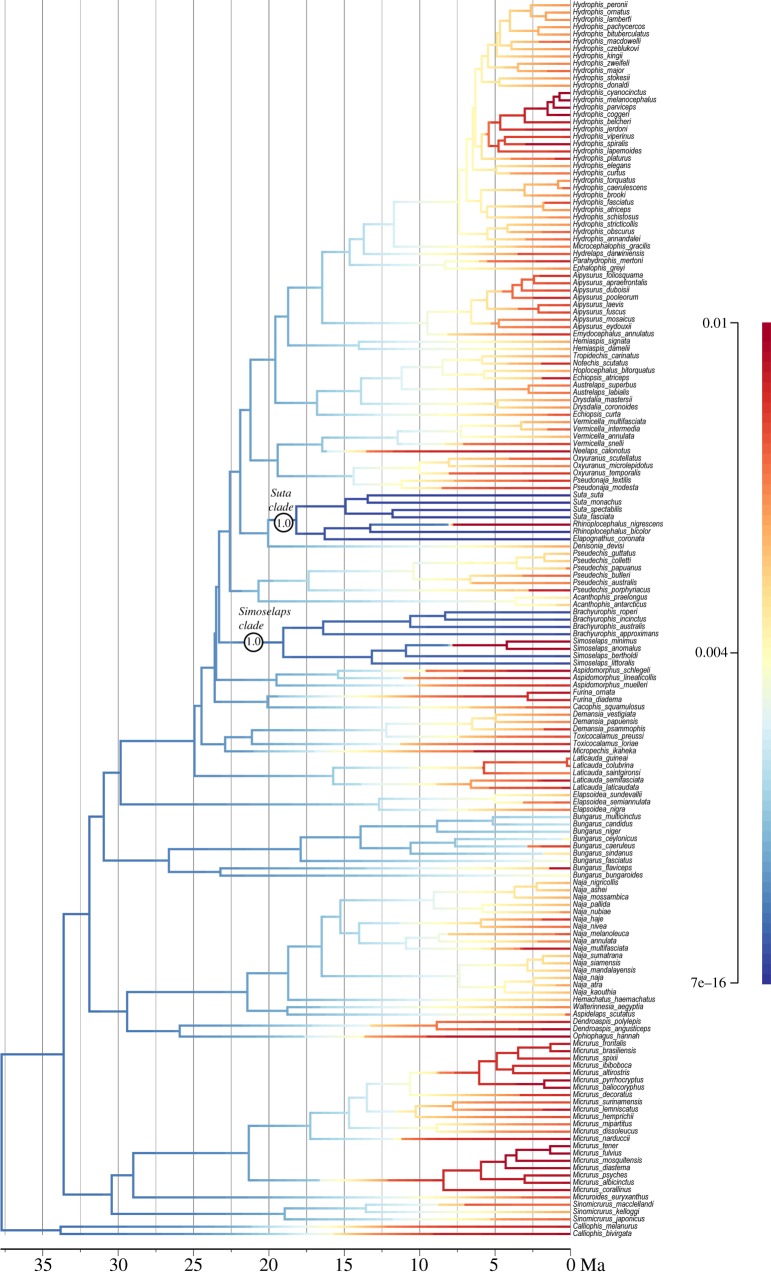
Datedphylogenetic tree of elapids, with rates of body size evolution inferred using BAMM (warmer colours = faster rates; scale bar on right in divergence per Ma); this is the same tree as figure 1 but with six taxa lacking size data excluded. Circled numbers indicate the two sizeable clades with the highest probability of rate shifts. Note that *Hydrophis* and *Micrurus* (figure 1) are not characterized by unusually fast rates of body size evolution. The three rate shift configurations (for size evolution) in the 95% credibility shift set are shown in electronic supplementary material, figure S7.

All analyses used four runs, each of four chains (incrementally heated at a coefficient of 0.04) with 50 million steps, sampling every 5000 steps. Convergence of numerical parameters (*ESS*>100 in Tracer [26]) and in topology (split frequencies less than 0.03) was achieved well before the burnin of 10 million (20%), and the post-burnin samples of all four runs were combined for summary statistics and a majority-rule consensus tree. This analysis resulted in a highly unbalanced (asymmetrical) consensus tree with *Calliophis* as sister to all other elapids (rather than related to other coral snakes, as proposed based on morphology (e.g. [27]) and from some molecular studies (e.g. [27,28])). However, because this separation was equivocal (*pp*=0.94), an analysis was also performed where coral snakes (*Calliophis*, *Micrurus*, *Micruroides* and *Sinomicrurus*) were constrained to be monophyletic, as supported by morphological data [27,29]. This resulted in a more balanced (symmetrical) tree. All diversification and phenotypic analyses were performed on the trees from both analyses, hereafter termed the ‘unconstrained’ (figures 1 and 2; electronic supplementary material, figure S1) and the ‘constrained tree’ (electronic supplementary material, figure S2). Both trees returned essentially identical results.

### 2.3 Phenotypic data

Data on body size for 169 of the 174 sampled species of elapid snakes in the molecular phylogeny were obtained from the literature (see list of species and sources in Dryad file 2). One of the major difficulties encountered while searching for size data resides in the inconsistent measurements provided by different sources (e.g. total length versus snout–vent length, average or typical length versus maximum length, length of females and/or males; mass was very rarely reported). Total (rather than snout–vent or tail) length was the most common value reported. Moreover, average (rather than maximum) total length was the most common value provided in the literature and is arguably a better index of a species’ size than is maximum total length (which is only representative of a few, perhaps atypical individuals). Average total length was thus used in this analysis as an index of size; in the rest of this paper, ‘length’ refers to ‘average total length’ unless otherwise specified. If not specified by the authors (all species in [30] and a few species in [14]), length measurements reported were assumed to be average. In the cases where both male and female lengths were provided, the average for both sexes was computed. Similarly, in cases where different authors provided different lengths for a given species, the average of these values was used.

For species where only maximum total length was provided in the literature, we statistically inferred the average total length using the following approach. The relationship between average total length and maximum total length was obtained for the 46 taxa where both variables were available (electronic supplementary material, figure S3). We then used this tight relationship (Log_10_[AveLength] = 1.00643 Log_10_[MaxLength] − 0.18503, *R*^2^=0.9 with 44 d.f.) to infer average length for those taxa in which only maximum length was provided; the 40 (of 169) species which had body lengths inferred in this fashion are identified in the electronic supplementary material, table S2. All data were log-transformed for analyses and available in the electronic supplementary material, file S2 (raw) and file S3 (logged).

### 2.4 Macroevolutionary analysis

Rates of diversification and evolution (average body length) were inferred on both the unconstrained tree and the constrained tree, using Metropolis-coupled Markov-chain Monte Carlo (MC3) as implemented in BAMM v. 2 [31], with relevant priors chosen using BAMMtools [32]. All settings used in the analyses are provided in the BAMM files (combined into Dryad file 3). All analyses were performed four times, and Tracer [26] was used to confirm these replicates converged on the same global optimum.

Speciation and extinction were inferred using the ‘speciation–extinction’ module, with correction for differential sampling across genera. The sampling fraction of each genus (electronic supplementary material, file S2) was determined by comparing the number sampled with the total number in the Reptile Database (www.reptile-database.org; accessed June 2014). Each run consisted of four chains, incrementally heated (temp 0.01) and 200 million steps long, with sampling every 40 000 steps; a burnin of 10% was found to be sufficient (see above).

Body size evolution was inferred using the continuous ‘trait’ module. These analyses reached convergence less readily, so each run consisted of eight chains, incrementally heated (temp 0.05) and 2000 million steps long, with sampling every 2 million steps, and a burnin of 50%. We used the full phenotypic dataset (169 species), as well as a conservative dataset including only the 129 species for which average adult size was directly obtained rather than inferred (see §2.3).

BAMMtools [32] was used for summary statistics, such as ‘phylorate’ plots (showing rates and rate shifts in diversification and size evolution), 95% credible sets of rate shifts configurations and average rates for the entire tree and particular clades. Branch-specific Bayes factors (BFs) [33] for each rate shift were calculated. These assess the posterior evidence for a rate shift on a branch relative to the prior probability, which is affected by both the prior on the total number of rate shifts and the relevant branch length. Phylogenetic generalized least squares, as implemented via Markov-Chain Monte Carlo in BayesTraits [34], was also used to confirm patterns that were inferred from visual inspection of the results (see below).

## 3. Results

### 3.1 Molecular phylogenetics

The dated consensus tree from the MrBayes analyses without any topological constraints (‘unconstrained tree’) is shown in figure 1, with clade probabilities shown in the electronic supplementary material, figure S1. The tree topology is highly unbalanced (asymmetrical), with *Calliophis* as sister to all other elapids. The dated consensus tree from the MrBayes analyses with coral snakes constrained to be monophyletic (‘constrained tree’) resulted in all coral snakes, rather than *Calliophis* alone, being sister to all other elapids (electronic supplementary material, figure S2). Topology was otherwise identical, and clade supports (posterior probabilities) and divergence dates were very similar. The tree broadly confirms the pattern of elapid relationships proposed by other recent studies, but refines certain phylogenetic arrangements and node dates (see Discussion).

### 3.2 Macroevolutionary analysis

Similar patterns of diversification and of body size evolution were retrieved from the BAMM analyses of both the optimal and the constrained tree; for brevity, the discussion focuses on the unconstrained tree (figure 1). Tables 1 and 2 summarize the results for both the unconstrained tree and constrained tree (electronic supplementary material, figure S2).

**Table 1.** Rates of speciation (λ) and extinction (*μ*) for selected clades of elapids, as inferred using the best tree (figure 1) and the constrained tree (electronic supplementary material, figure S2). The mean and upper/lower 95% highest probability density (HPD) for each estimate is presented. Bold denote clades with diversification regimes that were identified by BAMM as distinct from the elapid ‘background’: *Micrurus* and *Hydrophis* exhibit unusually rapid speciation and extinction rates. Brown, terrestrial taxa; blue, aquatic taxa.

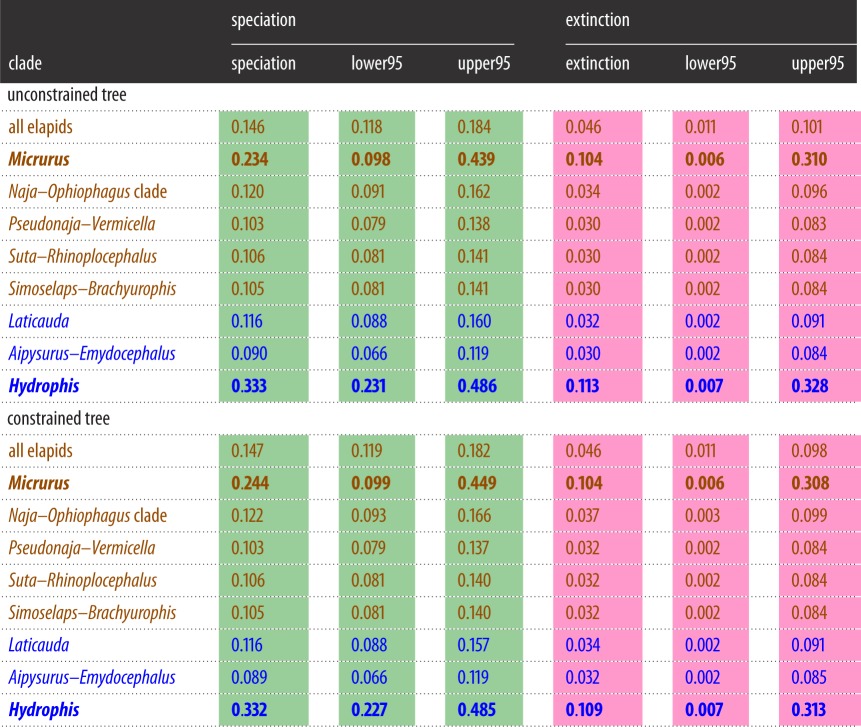

**Table 2.** Rates of size evolution (average total length) for selected clades of elapids, as inferred using the best tree (figure 1) and the constrained tree (electronic supplementary material, figure S2). The mean and upper/lower 95% HPD for each estimate is presented. Bold denote clades with diversification regimes that were identified by BAMM as distinct from the elapid ‘background’: *Suta–Rhinoplocephalus* (‘Suta clade’) and *Simoselaps–Brachyurophis* (‘*Simoselaps* clade’) exhibit unusually slow rates. Note that *Micrurus* and *Hydrophis* (which have exceptionally fast diversification rates (table 1)) have unremarkable rates of size evolution. Brown, terrestrial taxa; blue, aquatic taxa.

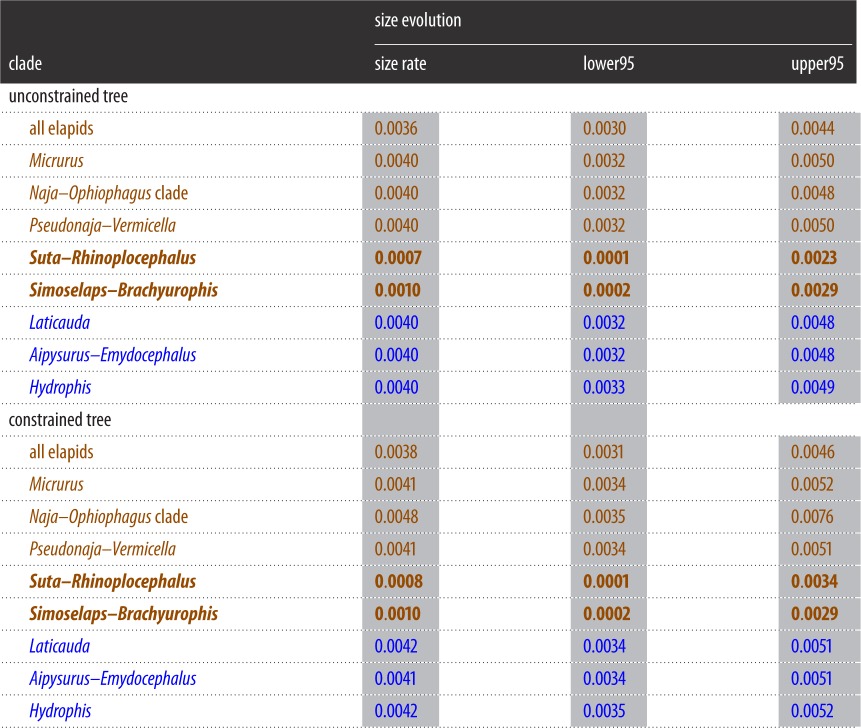

Across elapids as a whole, speciation rates generally decrease (figure 1) and extinction rates increase (electronic supplementary material, figure S4) through time, as would be expected given broadly density-dependent processes. However, two clades exhibit diversification regimes that stand out against this background: *Hydrophis* (=the core *Hydrophis* group [19]; regime shift probability 0.95) and *Micrurus* (probability 0.47); ‘raw’ posterior probabilities reported (not scaled relative to prior). These are also the clades with the highest branch-specific BF support for regime shifts, after correcting for the prior (8471 and 237, respectively). There are nine regime shift configurations that account for more than 95% of samples (electronic supplementary material, figure S5); all nine show shifts at either the stem or crown of *Hydrophis*, and five show shifts at either the stem or crown node of *Micrurus*. Average speciation and extinction rates for *Hydrophis* and *Micrurus* are more than double those of other elapid clades (table 1); this is due to a marked increase in rates in *Hydrophis*, and stable (rather than declining) rates in *Micrurus* (electronic supplementary material, figure S6). A model with two shifts in diversification regime had highest posterior probability (0.4) and was strongly supported over models with fewer regime shifts (BF>5.4), whereas models with more regime shifts did not fit appreciably better (BF<3).

Rates of body size evolution exhibited a very different pattern, which was retrieved with both the full or conservative body size dataset (see §2.4), so only the results with the full dataset are discussed. Nearly all elapids lineages share a single ‘background’ rate of body size evolution, with rates tending to increase slightly towards the present (figure 2). Two Australian clades stand out against this background in exhibiting very slow rates: smallish viviparous snakes (*Suta*+*Rhinoplocephalus*+*Elapognathus*) and tiny burrowers (*Simoselaps*+*Brachyurophis*). These are hereafter termed the ‘*Suta* clade’ and the ‘*Simoselaps* clade’, and each has a raw regime shift probability of 1.0; the BF support cannot be computed but is presumably high, as the prior probability (the divisor) appears to be rounded to zero in BAMM. There are three rate shift configurations that account for more than 95% of samples (electronic supplementary material, figure S7) and, as expected, all configurations show slowdowns at either the stem or crown of these two clades (with subsequent accelerations in one or two species). Average rates of body size evolution for *Suta* and *Simoselaps* clades are one-third those of other elapid clades, or even slower (table 2); due to constraints in BAMM, these calculated averages include the embedded ‘fast’ species and so are conservative (i.e. less extreme than if these embedded species could have been excluded).

In summary, rates of diversification were exceptionally fast in *Hydrophis* (0.333 speciation events per Ma, compared to the background rate of 0.129), but rates of size evolution were exceptionally slow in the *Suta*–*Rhinoplocephalus* and *Simoselaps*–*Brachyurophis* clades (0.0007 and 0.0010 change per Ma, respectively, compared to the background rate of 0.0039). Phylogenetic generalized least-squares analyses of these rates on the unconstrained phylogeny, as implemented in BayesTraits ([34]; data in Dryad File 4), confirmed lack of correlation: models with/without correlation parameter returned virtually identical marginal likelihoods (BF=0.28).

## 4. Discussion

It has often been suggested that elevated speciation rates might be correlated with increased rates of morphological change, and broad-scale phylogenetic evidence for this hypothesis has been found in some instances (e.g. [1,2,6]), but not others (e.g. [3–5]). We decided to test the relationship between speciation rates and changes in body size within Elapidae, a large and young group of venomous snakes whose members are extremely diverse in morphology, size, ecology and behaviour, and which therefore represent ideal candidates for this type of study (e.g. [10–13]).

We here generated the most densely sampled molecular phylogeny of elapid snakes to date, calibrated directly using fossils. The phylogeny (figure 1; electronic supplementary material, figure S1) is broadly consistent with previous studies (e.g. [18,28,35–37]). Basal lineages are predominantly Asian, suggesting that this was the distribution of the common ancestor of extant elapids. There is a ‘coral snake’ clade consisting of the Asian *Sinomicrurus* and the American *Micruroides* and *Micrurus*, and an Afro-Asian clade of cobras and mambas (*Naja*, *Hemachatus*, *Aspidelaps*, *Walterinnesia*, *Dendroaspis*, *Ophiophagus* and *Hemibungarus*). Kraits (*Bungarus*) are close relatives of a diverse clade that also includes all Australasian elapid genera, and sea snakes. Within the Australian radiation, the small burrowers form two distantly related clades, *Vermicella* and *Neelaps*, versus *Simoselaps* and *Brachyurophis.* Among relationships that have fluctuated in previous studies, the primitive Asian coral snake genus *Calliophis* (*Maticora*) emerges as monophyletic and sister to all other elapids, rather than paraphyletic [18] or closely related to other coral snakes [28]. The African garter snakes (*Elapsoidea*) are closely related to *Bungarus* and the Australasian clade [24,36], rather than part of the main Afro-Asian clade [28]. Three elapid species are included in molecular phylogenies for the first time. *Hydrophis bituberculatus* emerges as sister to *H. pachycercos*, and *H. torquatus* emerges as sister to *H. caerulescens*, with strong support (electronic supplementary material, figure S1). The poorly known sea snake genus *Kolpophis* (consisting of the sole species *K*. *annandalei*) emerges as nested within *Hydrophis* (being weakly resolved as sister to *H. obscurus* and *H. stricticollis*) and for the rest of the discussion is considered part of *Hydrophis*, along with several other monotypic genera that were also recently identified as part of the core *Hydrophis* clade [19].

The analyses retrieved a relatively recent time-frame for elapid diversification, broadly consistent with most recent studies (e.g. [18,24,37]). The crown elapid radiation is approx. 38 Myr old, and (core) coral snakes, the (core) Afro-Asian clade and Australasian clade, including *Bungarus* and *Elapsoidea*, all diversified around 30–25 Ma. The Australasian clade (Hydrophiinae) is approximately 25 Myr and viviparous sea snakes (Hydrophiini) are approximately 16 Myr, with *Hydrophis* being only approximately 8 Myr old.

This phylogeny reveals no correlation between increased diversification rates and accelerated rates of body size change (a common proxy for morphological variation). Notably, the two most rapidly speciating clades (*Micrurus* coral snakes and *Hydrophis* sea snakes: figure 1 and electronic supplementary material, figure S6) show virtually identical rates of body size evolution to other elapids (figure 2). However, rates of diversification could still be correlated with rates of change in phenotypic traits other than body size. Another possibility is that there is an association that was not evident due to insufficient data, but the relatively large phylogeny (approx. 170 species) used here and the lack of correlation in the two traits suggest that any such association must be extremely weak at best. *Hydrophis* is the prime outlier for fast speciation rates, and there are several plausible reasons behind this. First, the clade exhibits exceptional ecological diversity, especially relating to prey preference (e.g. [38]), and it also collectively has a wide distribution that extends far beyond the range of other sea snake groups. Thus, speciation might be facilitated by a combination of (i) an intrinsic propensity to exploit ecological opportunities for diversification; (ii) geographical ranges that encompass broad, ecologically heterogeneous marine regions, which in combination with ecological barriers generated by sea-level changes in the last 2.5 Myr may have accelerated rates of both allopatric and ecological speciation [39]; and (iii) a lack of ecological analogues (potential competitors, including species from the *Aipysurus*–*Emydocephalus* clade) throughout much of its range. Finally, fast ‘early burst’ speciation rates operated relatively recently within *Hydrophis*, with the patterns less heavily overwritten by a subsequent period of declining probability of speciation and/or lineage persistence [40]. However, the uniqueness of *Hydrophis*, especially regarding (ii) and (iii), means there is lack of phylogenetic replication within elapids, and indeed snakes, to rigorously test these explanations. As noted previously [41], invasion of a new biome (marine habitat) cannot be an explanation by itself, as the marine relatives of *Hydrophis* have rates of speciation similar to terrestrial elapids. Moreover, fairly typical speciation rates are found in a separate marine radiation, the sea kraits (*Laticauda*).

It is more equivocal whether there are elevated speciation rates in *Micrurus* (see above); if one accepts this pattern, the drivers are less obvious. Limited competition from ecological analogues might be a potential factor. *Micrurus* is the only sizeable radiation of burrowing venomous snakes in the New World [42,43]; the only other analogues consist of the monotypic *Micruroides* (which is the sister lineage to *Micrurus* and has a very limited geographical distribution), and the colubrid *Phalotris*. In contrast, there are two burrowing lineages in Australasia (*Vermicella*+*Neelaps*, *Simoselaps*+*Brachyurophis*) [27]. However, the combined species diversity (approx. 15 species) of all burrowing forms in Australia is still far less than *Micrurus* (approx. 79 species; www.reptile-database.org, June 2015). *Micrurus* also shows high variability in number of chromosomes [43], which are thought to have undergone numerous inversions and fissions [44]. This may be linked with a putatively higher speciation rate [45], though comparative data across all elapids is required.

Rates of size evolution were identified as unusually slow in the *Simoselaps* clade and in the *Suta* clade (electronic supplementary material, figure S2). Again, there is a plausible driver for this pattern in one of these clades. A possible reason for the conserved body size in the *Simoselaps* clade relates to its antiquity and specializations. The *Simoselaps* clade is the oldest of the two burrowing clades in Australia and comprises those elapids that are most adapted for a fossorial existence: for instance, *Brachyurophis* is characterized by a shovel-like upper lip (rostral scale), while *Simoselaps* exhibits sand-swimming. The biomechanics of burrowing and subterranean locomotion impose strong constraints towards small body size (i.e. reduced diameter) in limb-reduced reptiles (e.g. [46]). Fossorial limbless reptiles (e.g. amphisbaenians, dibamids, various scincids, blindsnakes) are typically much smaller than most advanced snakes. That the *Simoselaps* clade includes most of the smallest elapids in our sample (the two smallest, and eight of the 11 smallest) is consistent with this trend. Thus, it is conceivable that the *Simoselaps* clade reached the lower size limits possible for elapids some time ago, and has since remained stable in size. In contrast, the other burrowing clade, the *Vermicella* clade, is younger, retains species with larger size and is less specialized for a fossorial existence.

The reasons for the apparent stability of body size in *Suta* are less obvious, given that they appear to be fairly typical members of the Australian ‘viviparous clade’ in most respects. One possible explanation could reside in the observation that gravid females of *Suta* may become extremely secretive, more so than typical elapids. It has been reported [47] that only about 3% of the adult females in museum collections are gravid specimens (compared to more than 10% for other viviparous Australian elapids). Small size would be advantageous for animals that periodically need to hide in burrows or crevices, and this may impose a constraint on their maximum body size.

The diversification rates show a remarkable consistency across most of the phylogeny, with the Asian, African and Australasian radiations sharing a broadly similar rate regime. There does not appear to be any evidence for rapid lineage or body size diversification coinciding with the colonization of new continents, in contrast to the pattern suggested for other snake groups such as pythons [48]. While Australasian elapids contain more species than other continents, much of this imbalance is due to one highly atypical clade of viviparous sea snakes (*Hydrophis* accounts for approx. 30% of species richness in this group). Similarly, taxa from all continents share a broadly similar rate of body size evolution, which is reduced in the *Suta* and *Simoselaps* clades. A possible explanation is that the rapid worldwide diversification of elapids, with all continents colonized within a period approximately between 30 and 25 Ma, is adequately modelled by a single rapid diversification rate regime (e.g. [33]). Nevertheless, it is surprising that the colonization of Australasia by elapids did not result in sharply increased rates of speciation or phenotypic evolution: this continent differed greatly from all other continents in lacking other advanced snakes, i.e. caenophidians [49]. A study across snakes in general [13] similarly found no evidence for elevated rates of body shape change at the base of the Australasian elapid radiation.

The lack of association between elevated speciation rates and body size evolution, and between these two evolutionary processes and the colonization of Australasia, is surprising. The lack of association between diversification and/or trait evolution with candidate evolutionary drivers has been noted (e.g. [13,33]) and might reflect a widespread pattern. Alternatively, it might be an artefact of our reduced power to detect macroevolutionary patterns in the deep past using (primarily) extant information, especially for groups with a poor fossil record, including elapids [7,50,51].

These results highlight the complexity of teasing apart patterns of evolution across large evolutionary trees. Certain patterns (e.g. rapid speciation rates in *Micrurus*, slow body size evolution in *Suta*) might be difficult to explain because they are based on rare or unique combinations of causal factors. A possible solution would be to analyse larger and larger phylogenies, where enough data might tease apart multiple causes and where even rare causal combinations might be replicated [3]. This is one of the most promising ways forward, especially given the increasing availability of such mega-phylogenies (e.g. [18,36,52–54]). However, such analyses are typically forced to make assumptions about the homogeneity of processes across vast expanses of the tree of life, and these assumptions can be problematic. For instance, if speciation is correlated with body size evolution in one clade but not another, an analysis of the global phylogeny testing for a single overall pattern would inevitably give a misleading answer. Understanding the subtleties and complexities of biological evolution therefore requires both large-scale and smaller scale comparative studies.

## Supplementary Material

FigsS1-S7_TabS1-2.docx - All Supplementary Figures and Tables.

## Data accessibility

The supplementary tables and figures supporting this article have been uploaded as part of the electronic supplementary material, and electronic supplementary material S1–S4 are on the Dryad digital repository (http://dx.doi.org/10.5061/dryad.cr788).
