# Supplementary material for: Diversification rates and phenotypic evolution in venomous snakes (Elapidae)
Source: R Soc Open Sci. 2016 Jan 20;3(1):150277. doi: 10.1098/rsos.150277 (PMC4736917; doi:10.1098/rsos.150277)

Table S1. New taxa and sequences added to the alignment in Pyron et al. (2013), to generate the matrix in File S1. Shaded cells indicate taxa included based on loci and sequences from Sanders et al. [19]. Genbank numbers for new sequences from this study are highlighted in red.

	16S	12S	ND4	cytb	cmos	RAG1
Aipysurus_foliosquama	KU240026			KP205509		
Aipysurus_mosaicus						
Aipysurus_pooleorum						
Brachyurophis_approximans	KF736406	KF709658				
Brachyurophis_australis	KF736383	KF709663				
Brachyurophis_incinctus	KF736319	KF709668				
Brachyurophis_roperi	KF736376	KF709666				
Hydrophis_(Kolpophis)_annandalei	KU240027		KU240028	KU240025		
Hydrophis_belcheri						
Hydrophis_bituberculatus			KU240029			
Hydrophis_coggeri						
Hydrophis_donaldi	JQ217147		JQ217211	JQ217202	KC014308	KC014285
Hydrophis_fasciatus						
Hydrophis_jerdoni						
Hydrophis_lamberti						
Hydrophis_obscurus						
Hydrophis_ocellatus						
Hydrophis_pachycercos						
Hydrophis_stricticollis						
Hydrophis_torquatus			KU240030			
Hydrophis_viperinus						
Hydrophis_zweifeli	JX987143		JX987173	JX987165	JX987183	JX987177
Laticauda_semifasciata				AB701344		
Microcephalophis_gracilis						
Micrurus_mosquitensis			JF308712			
Micrurus_spixii			JF308708			
Micrurus_tener			JF308711			
Oxyuranus_temporalis			JN834014			
Simoselaps_littoralis	KF736320	KF709683				
Simoselaps_minimus	KF736359	KF709688				
Vermicella_annulata	KF736401	KF709677				
Vermicella_multifasciata	KF736403	KF709678				
Vermicella_snelli	KF736400	KF709675				

Table S2. The four calibration points used in the dated phylogenetic analysis, enforced as offset exponential distributions with the hard minima (offset) and soft maxima (encompassing 95% of distribution). The MrBayes commands are in File S1.

**Node**	**Hard Minimum**	**Soft 95% Maximum**	**Comments**
Elapidae	24.95	37.2	Hard Min: The oldest crown elapid vertebrae are 24.95 Ma (McCartney et al. 2014), with one taxon having similarities to asian elapids and another taxon similarities to African elapids. Other elapids (but not definitely crown) are known from 20-25Ma (Rage 1988). Soft Max: Numerous caenophidians, including colubroids, are present in the deposits that are ~34 Ma, ~35Mw and 37.2Ma (Head et al. 2005; Sanders et al. 2008), but no potential elapids (stem or crown) have been reported, suggesting they were absent during this interval.
Hydrophiinae (*sensu lato*)	16	26	Hard Min: The oldest Hydrophiinae - Australasian terrestrial and marine elapids - is from RSO site in North Queensland (Scanlon et al. 2003) which is 16-23 Ma (Travouillon et al. 2006). Soft Max: There are no elapids known from all earlier sites in Australia, which span ages of 24-26Ma, so elapids were likely, but not definitely, absent during this interval.
Naja_Hemachatus clade	18	32	Hard Min: *Naja romani* is known from MN4, middle Orleanians (~18Ma: Ivanov 2002). It has a few synapomorphies with asiatic *Naja* (suggesting it belongs to crown *Naja*), but retains some primitive traits (rare/absent in living *Naja*) that conversely suggest it is a stem *Naja* (Szyndlar and Rage 1990). Until this is resolved, we take the conservative view and assume this species is at least total group *Naja* stem or crown, and thus at least dates the split between *Naja* and it's nearest relative. Soft Max. Numerous deposits down to early Oligocene in Europe and North America (~32 Ma) have yielded a rich fauna of colubrids, including unidentified elapids 20-25 Ma, but nothing assignable to modern genera such as *Naja* (Rage 1988).
Micururus_Sinomicrurus	18	32	Hard Min: *Micrurus gallicus* is known from MN4, middle Orleanians (~18Ma: Ivanov 2002); it has similarities with extant *Micrurus* (Szyndlar and Schleich 1993). Soft Max. Numerous deposits down to early Oligocene in Europe and North America (~32 Ma) have yielded a rich fauna of colubrids, including unidentified elapids 20-25 Ma, but nothing assignable to modern genera such as *Micrurus* (Rage 1988).

Head J., Holroyd P.A., Hutchison J.H., Ciochon R.L. 2005. First report of snakes (Serpentes) from the late middle Eocene Pondaung formation, Myanmar. *J. Vert. Paleontol.* 25, 246–250.

Ivanov M. 2002. The oldest known Miocene snake fauna from Central Europe: Merkur–North locality, Czech Republic . Acta Palaeontologica Polonica 47, 513-534.

McCartney JA, Stevens NJ, O'Connor PM. 2014. The earliest colubroid-dominated snake fauna from Africa: perspectives from the Late Oligocene Nsungwe Formation of Southwestern Tanzania. *Plos ONE* 9(3): e90415. doi:10.1371/journal.pone.0090415.

Rage JC. 1988. The oldest known colubrid snakes: state of the art. *Acta Zool. Cracov.* 31:457–474.

Sanders KL, Lee MSY. 2008. Molecular evidence for a rapid late- Miocene radiation of Australasian venomous snakes (Elapidae: Colubroidea). Mol. Phylogenet. Evol. 46, 1180–1188.

Scanlon JD, Lee MSY, Archer M. 2003. Mid-Tertiary elapid snakes (Squamata, Colubroidea) from Riversleigh, northern Australia: early steps in a continent-wide adaptive radiation. *Geobios* 36, 573– 601.

Szyndlar Z, Rage JC. 1990. West Palearctic cobras of the genus *Naja* (Serpentes:Elapidae): interrelationships among extinct and extant species. *Amphibia-Reptilia* **11**:385-400

Szyndlar Z, Schleich HH. 1993. Description of Miocene snakes from Petersbuch 2 with comments on the Lower and Middle Miocene ophidian faunas of Southern Germany. *Stuttgarter Beitr.ge zur Naturkunde Ser. B* 192,1–47.

Travouillon KJ, Archer M, Hand SJ, Godthelp H. 2006. Multivariate analyses of Cenozoic mammalian faunas from Riversleigh, northwestern Queensland. *Alcheringa Special Issue 1,* 323-349.

Fig. S1. "Best Tree": majority-rule consensus tree from the unconstrained MrBayes analysis ("best tree"). Posterior probabilities of clades are indicated, and timescale is in millions of years.

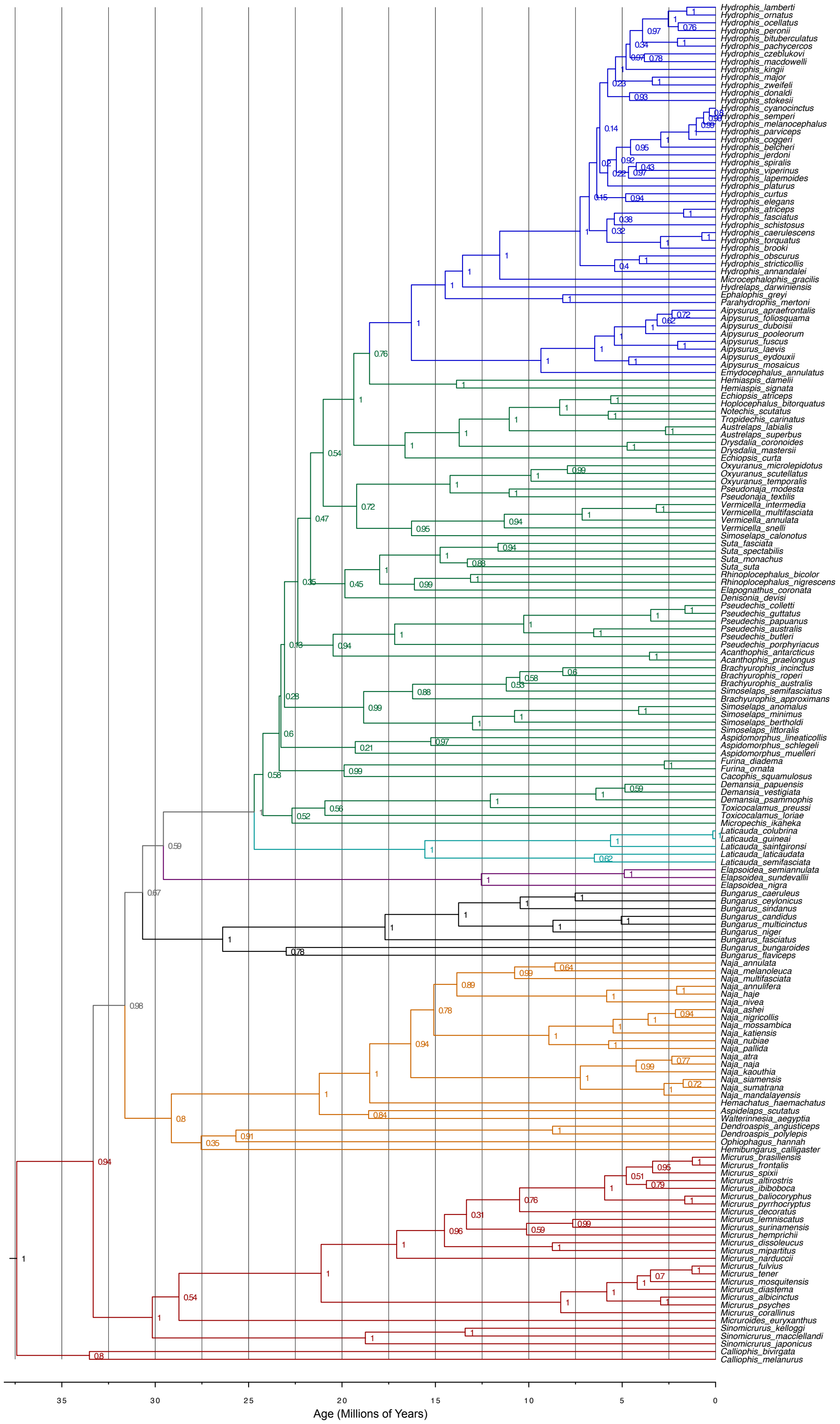

Fig. S2. "Alternative Tree": majority-rule consensus tree from the MrBayes analysis where coral snakes (red) are constrained to be monophyletic. Posterior probabilities of clades are indicated, and timescale is in millions of years.

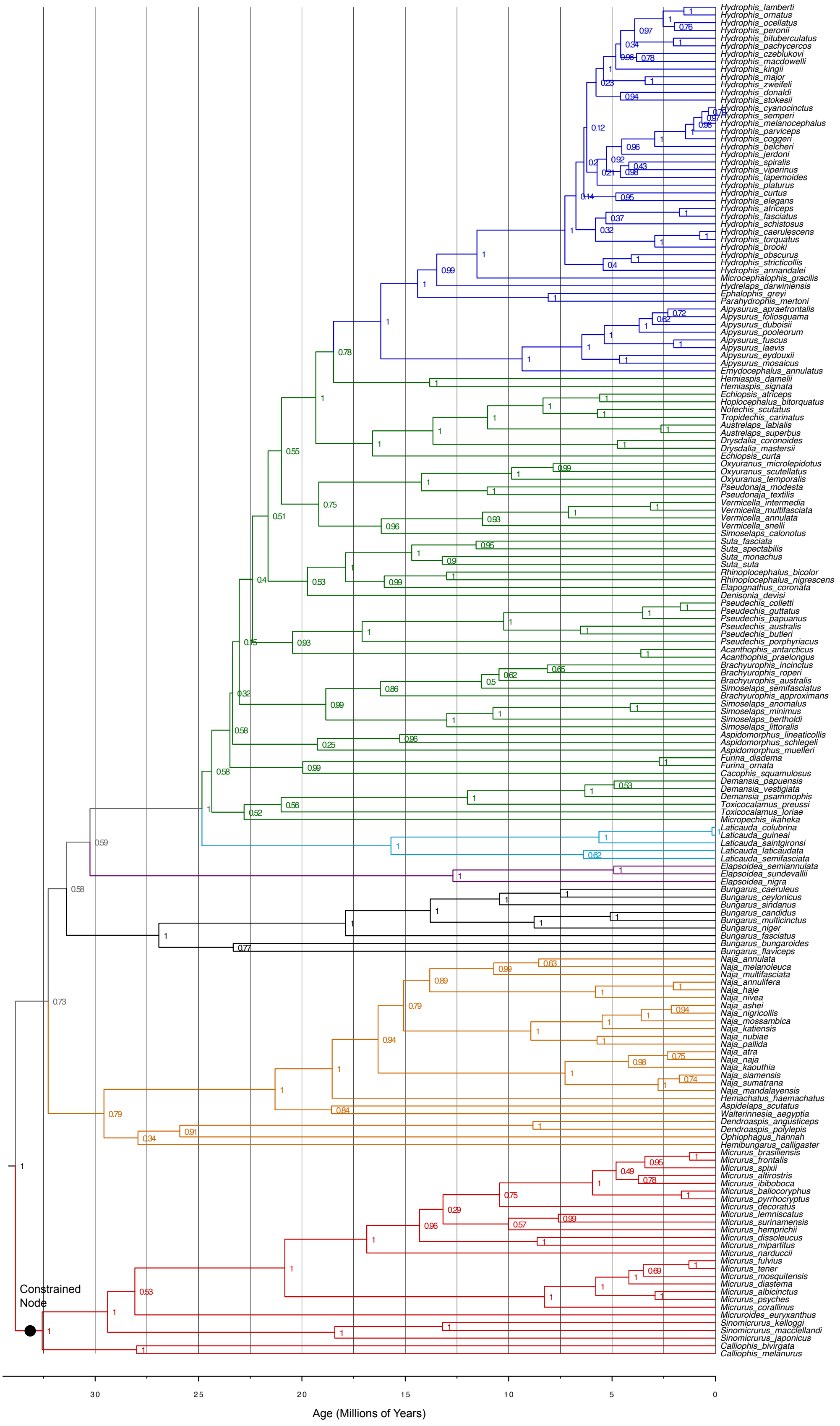

Fig. S3. Relationship between maximum length and average length in 46 species of elapid snakes where both measurements were available (File S2). LS regression (for predicting Ave length using Max length): Log_{10}[AveLength] = 1.00643 Log_{10}[MaxLength] - 0.18503, R^2 = 0.9 with 44 d.f.

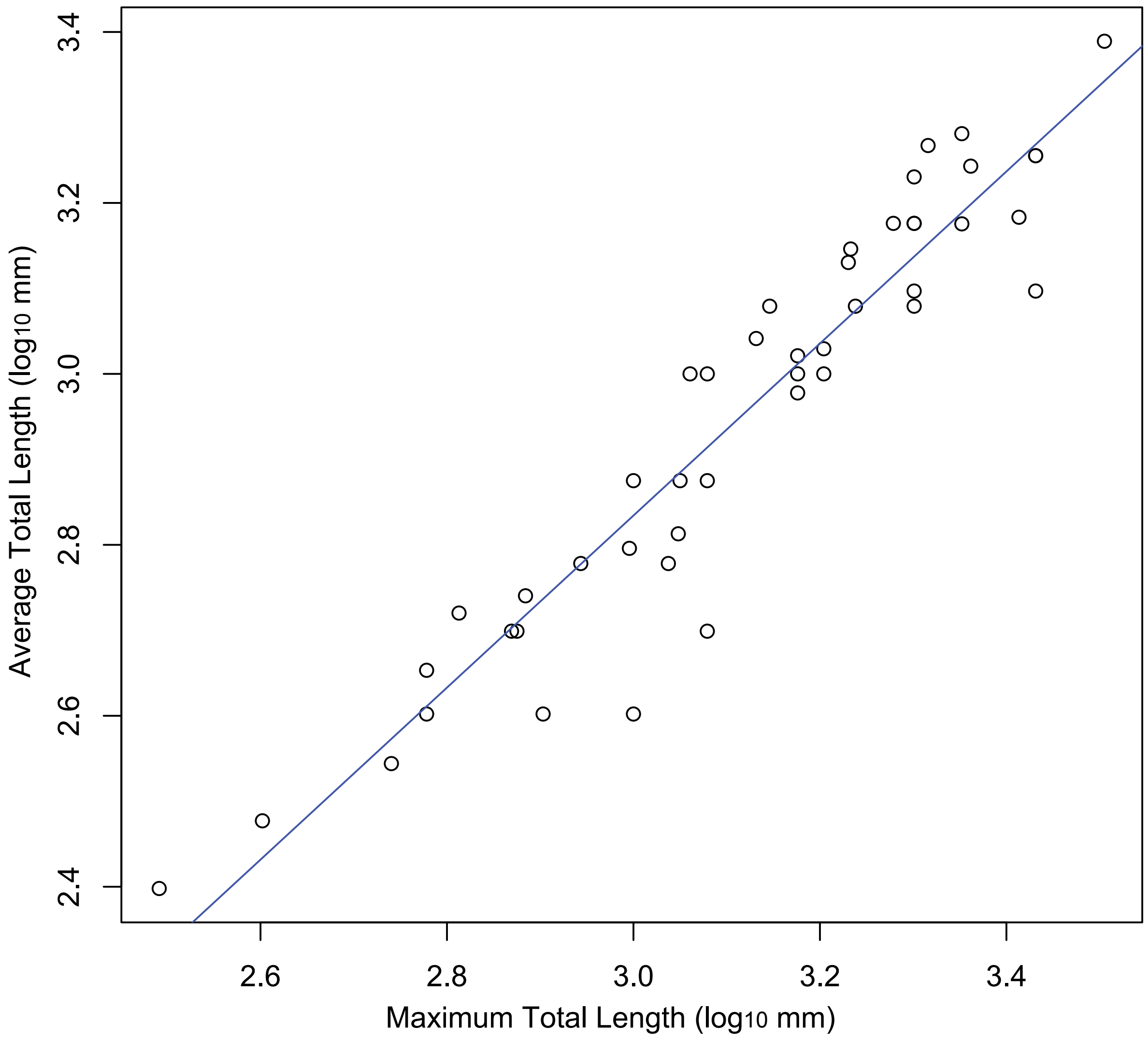

Fig. S4. Dated phylogenetic tree of elapids with rates of extinction inferred using BAMM (warmer colours = faster rates). Circled numbers indicate the two branches with the highest probability of rate shifts, i.e. *Hydrophis* and *Micrurus*. Tree with posterior probabilities of each clade is shown in Fig. S1, rates of speciation are shown in Fig. 1, and the nine rate shift configurations (for diversification) in the 95% credibility shift set are shown in Fig. S5.

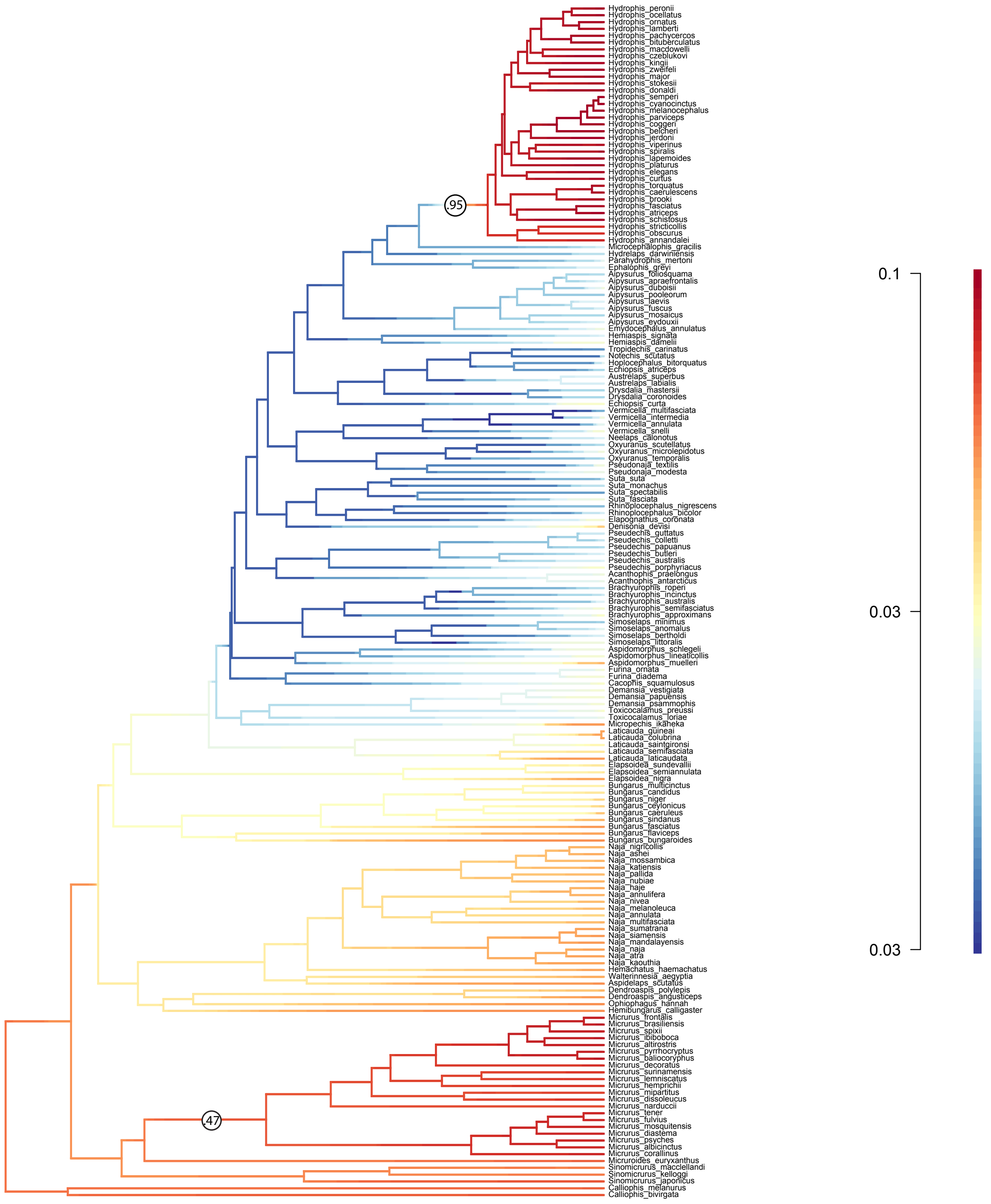

Fig. S5. The 9 most frequently sampled rate shift configurations for speciation/extinction for tree S1, which together comprise the 95% credibility set, as extracted using BAMMtools. Warmer colours = faster rates; size of circle indicates posterior probability of shift (across all sampled trees); red circles denote increases in rate (at initiation of the new regime), blue circles denote decreases. Note that the rate in the new regime in *Micrurus* is initially slightly <u>lower</u> than the background regime in some configurations (1, 7 and 8), but this initial rate is maintained in *Micrurus* (whereas the background rate declines rapidly), resulting in higher overall speciation rates for *Micrurus;* see Fig. S6.

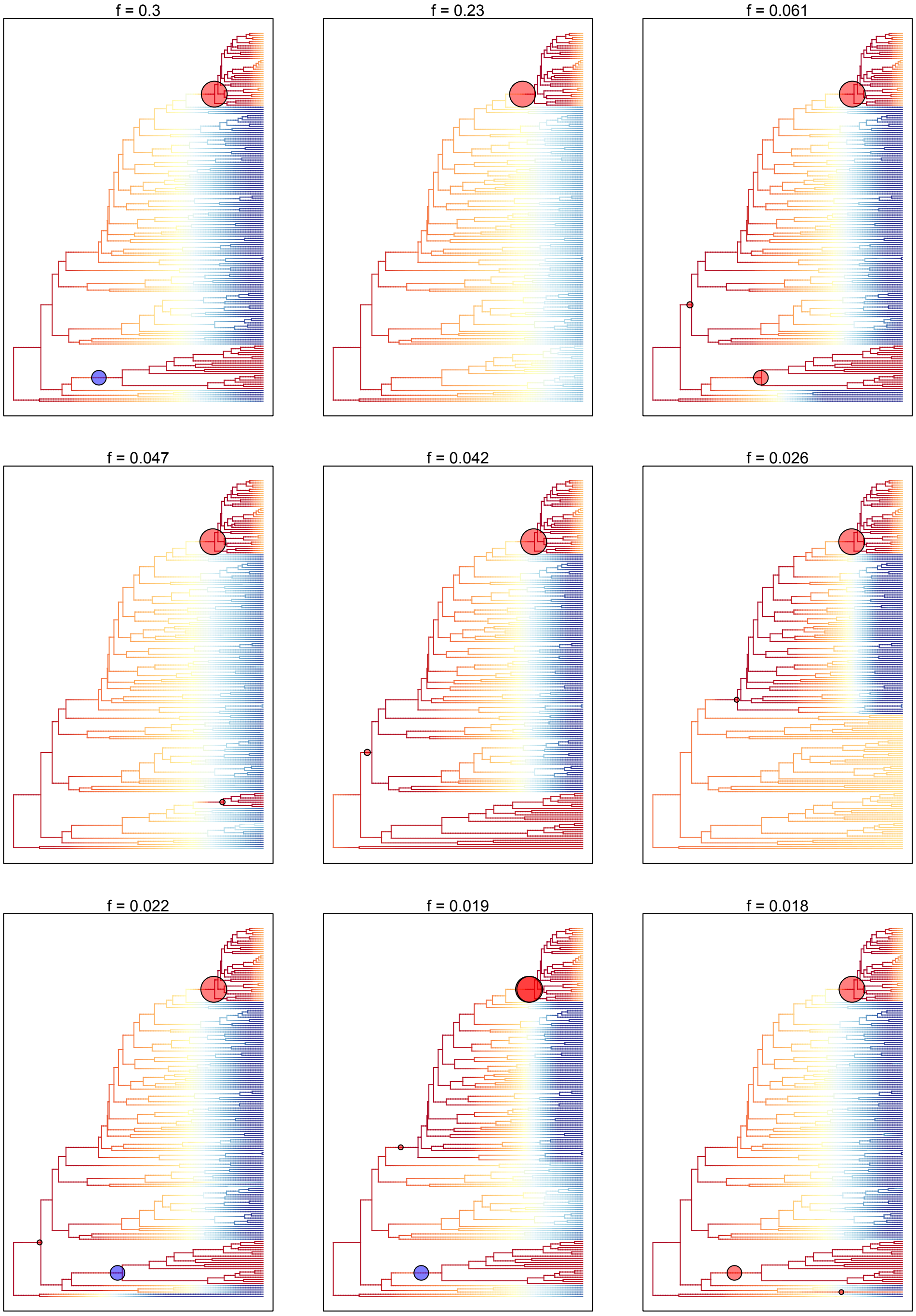

Fig. S6. Speciation rates for elapids in general (excluding *Hydrophis*), *Hydrophis,* and *Micrurus.* Speciation rates decline with time for elapids in general, with two regime shifts: an abrupt increase and subsequent decline in *Hydrophis*, and a shift to a stable rate in *Micrurus.*

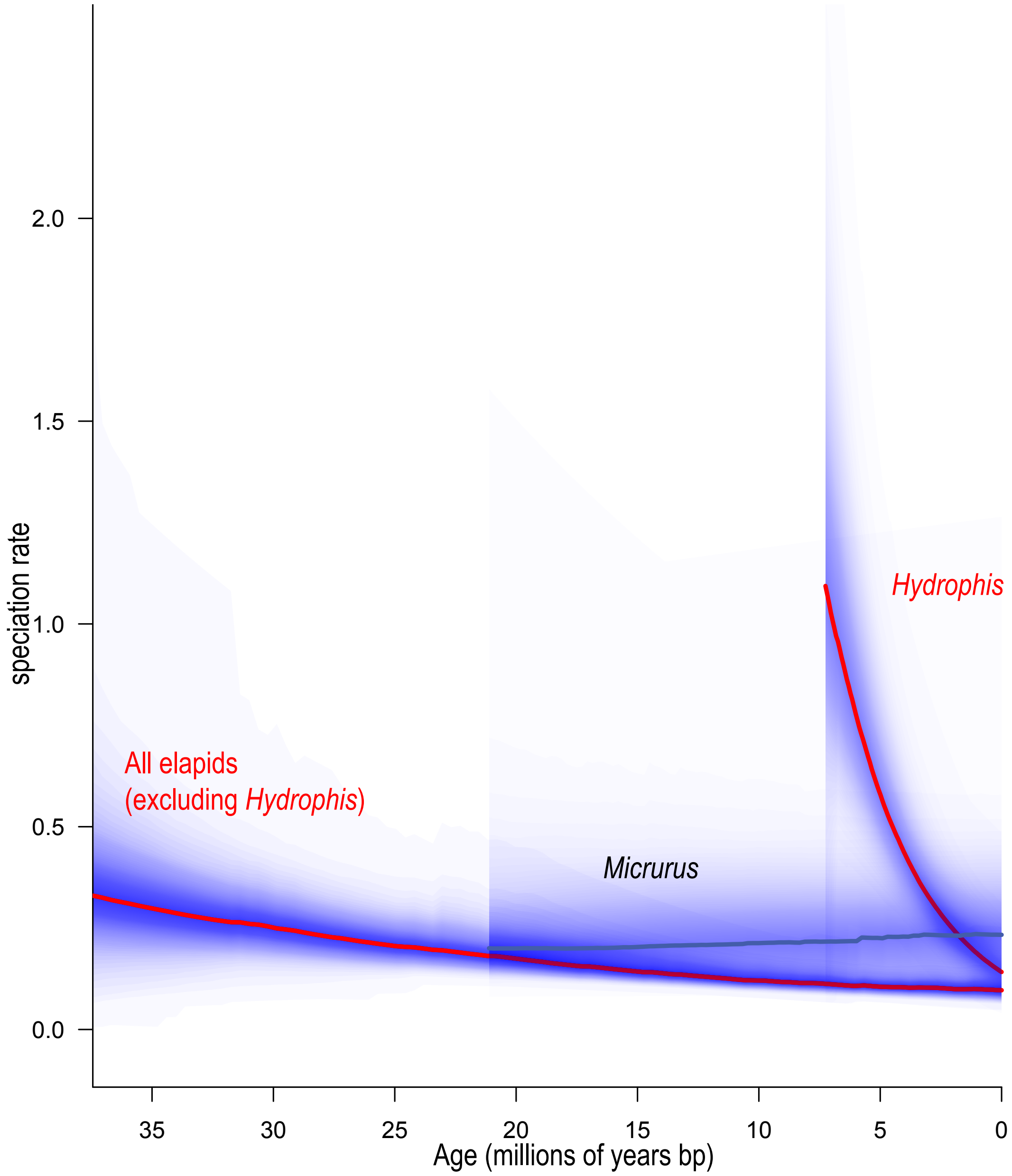

Fig. S7. The 3 most frequently sampled rate shift configurations for evolution of body size (average total length) for tree S1, which together comprise the 95% credibility set, as extracted using BAMMtools. Blue circles indicate decelerations, red circles accelerations, and size of circle is proportional to magnitude of shift. Warmer colours = faster rates; size of circle indicates posterior probability of shift (across all sampled trees); red circles denote increases in rate (at initiation of the new regime), blue circles denote decreases.

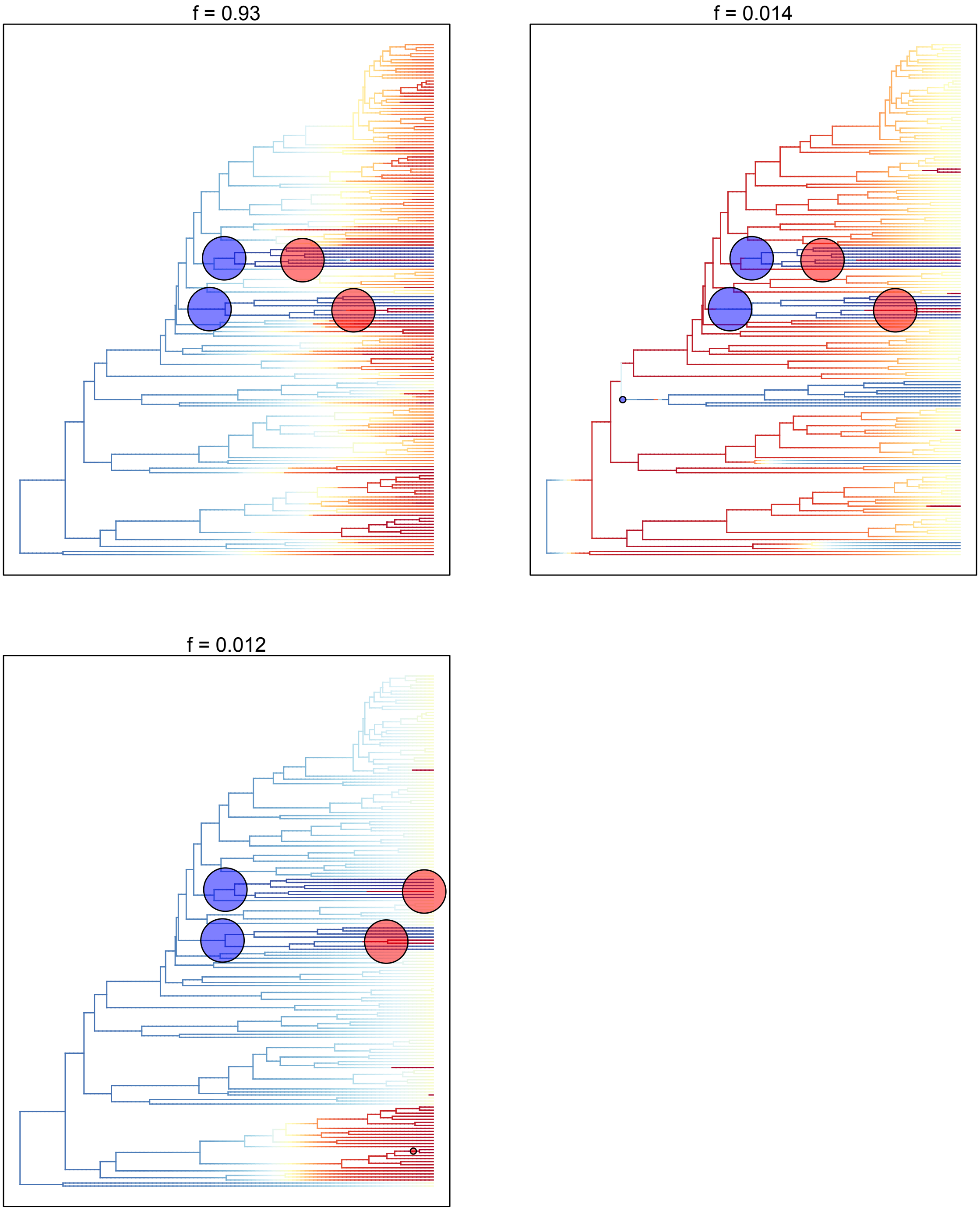

Supplement: FigsS1-S7_TabS1-2.docx - All Supplementary Figures and Tables. [file rsos150277supp1.pdf]
